# Study of the genetic and molecular epidemiology of cystic fibrosis based on the patient registry for planning targeted therapy in Russian Federation

**DOI:** 10.3389/fgene.2024.1383033

**Published:** 2024-10-28

**Authors:** Elena Kondratyeva, Yuliya Melyanovskaya, Victoriya Sherman, Anna Voronkova, Elena Zhekaite, Stanislav Krasovsky, Elena Amelina, Nataliya Kashirskaya, Vera Shadrina, Alexander Polyakov, Tagui Adyan, Olga Sсhagina, Marina Starinova, Elena Enina, Andrey Vasilyev, Andrey Marakhonov, Rena Zinchenko, Sergey Kutsev

**Affiliations:** Research Centre for Medical Genetics, Moscow, Russia

**Keywords:** cystic fibrosis, genetic epidemiology, CFTR gene variants, targeted therapy, disease prevalence

## Abstract

Cystic fibrosis (CF) is a genetically inherited disorder characterized by a wide range of clinical manifestations and genetic variations. This study focuses on the genetic and molecular epidemiology of CF in the Russian population, utilizing data from the national CF registry. The birth prevalence of CF in Russia has been analyzed over a span of years, revealing variations in frequency. The study delves into the genetic landscape of *CFTR* gene variants in Russian patients, showcasing a diverse spectrum with a predominance of severe variants, some of which are rare and distinct from global populations. A total of 233 variants have been documented, exhibiting frequencies ranging from 0.01% to 51.5%, with 47 of these variants remaining uncharted within international genetic databases. As of 2021, CFTR modulator therapy has been introduced for patients under 19 years, heightening the importance of genetic diagnosis. In 2023, more than 1,850 patients under 19 received CFTR modulator therapy. Notably, the impact of complex alleles on disease progression and response to targeted therapies is gaining recognition. Comparisons with European registries highlight distinctive features of the Russian population, such as differences in age distribution among patients. Additionally, the study emphasizes the need to ascertain clinical significance and pathogenicity of newly identified genetic variants, along with exploring their suitability for targeted therapies. The integration of genetic insights into the management of CF offers potential for enhanced personalized therapeutic interventions. In conclusion, this thorough analysis provides a comprehensive understanding of the genetic nuances within the Russian CF population. By illuminating the intricate relationship between genetic variations and disease manifestation, the study underscores the essential role of genetics in shaping therapeutic strategies and improving patient outcomes. Further research and ongoing genetic exploration are crucial for optimizing the care of individuals with CF in the era of evolving therapeutic options.

## 1 Introduction

Cystic fibrosis (CF; OMIM #219700) is an autosomal recessive disorder caused by pathogenic variants in the nucleotide sequence of the *CFTR* gene (OMIM *602421). The clinical presentation of CF varies widely, ranging from mild monosymptomatic manifestations to severe multi-organ involvement (Mall et al., 2024; Grasemann and Ratjen, 2023). In European countries, the average prevalence of CF is 1 in 7,000 newborns (Castellani et al., 2018), while in the Russian Federation, it ranges from 1 in 8,000 to 1 in 10,000 newborns, showing year-to-year fluctuations and variation among populations and federal districts (Kashirskaya et al., 2021).

The spectrum and frequency of pathogenic variants in the *CFTR* gene exhibit significant diversity across different populations and ethnic groups. To date, over 2000 genetic variants of the *CFTR* gene have been identified, of which 719 pathogenic variants are cataloged on the CFTR 2 international project website (https://cftr2.org) as of 2023. Utilizing data from the federal register, genetic and molecular epidemiology studies not only help elucidate potential variations in CF prevalence among different federal districts of the Russian Federation and distinguish prevalent genetic mutations from novel ones, but also offer insights into the prospects of CFTR modulator prescriptions for patient care.

This study aims to investigate the genetic and molecular epidemiology of cystic fibrosis within the Russian population, utilizing epidemiological data and information from the 2021 registry. By analyzing epidemiological data and leveraging the 2021 CF patent Registry (RCFPR), this research seeks to provide a comprehensive understanding of the genetic and molecular aspects of cystic fibrosis in the Russian population. This investigation will contribute to a deeper comprehension of the prevalence, distribution, and molecular variations of the disease’s genetic mutations among different regions and demographic groups within Russia. Additionally, the study intends to identify common genetic variants as well as previously unreported mutations, shedding light on the disease’s genetic landscape in this specific population. Ultimately, the findings of this research hold the potential to advance our knowledge of cystic fibrosis in the Russian population, paving the way for more accurate diagnosis, better targeted treatment approaches, and potential advancements in therapeutic interventions.

## 2 Materials and methods

The birth prevalence rates of individuals with cystic fibrosis (pwCF) between 2007 and 2022 were meticulously investigated by analyzing data from the federal register. This analysis involved calculating the ratio of identified CF patients for a specific year to the total number of newborns during that same year. The data source for this comprehensive study was the website of the federal register, available at (https://amg-genetics.ru/) (Krasovsky et al., 2021).

The research process encompassed the collection and examination of information from various regions within the Russian Federation (RF). The focal point was the meticulous analysis of the Register of Patients with Cystic Fibrosis in Russia for the year 2021. This database is a compilation of data from all regions that are considered constituent entities of the Russian Federation, with the exception of the Nenets and Chukotka Autonomous Districts. These two districts were not included due to official records from the Ministry of Health of the Russian Federation, which indicate the presence of only one CF patient within these areas.

Furthermore, special attention was given to the prominent urban centers of Moscow and St. Petersburg. The data related to these cities were treated separately, and specific indicators were presented for each. For a more comprehensive overview, Table 1 has been provided, offering insight into the population figures and the corresponding numbers of CF patients. The information is categorized according to the relevant Federal Districts, providing a comprehensive view of the distribution of patients across the Russian regions.

**TABLE 1:** Information regarding population and the number of CF patients, categorized by Federal Districts.

District	Population size	Number of patients, n (%)			p (between adults and children)
		Adults	Children	Total	
Central Federal District	39 250 960	374 (31.72%)	805 (68.28%)	1,179	*р* < 0.001
Northwest Federal District	13 941 959	88 (25.07%)	263 (74.93%)	351	*р* = 0.298
Southern Federal District	16 482 488	103 (24.18%)	323 (75.82%0	426	*р* = 0.111
Volga Federal District	29 070 827	259 (31.39%)	566 (68.61%)	825	*р* = 0.004
Ural Federal District	12 329 500	80 (24.61%)	245 (75.39%)	325	*р* = 0.234
Siberian Federal District	17 003 927	97 (21.13%)	362 (78.87%)	459	*р* = 0.001
Far Eastern Federal District	8,124 053	58 (28.43%)	146 (71.57%)	204	*р* = 0.744
North Caucasian Federal District	9,967 301	30 (15.00%)	170 (85.00%)	200	*р* < 0.001
Moscow	12 655 050	170 (37.36%)	285 (63.64%)	455	*р* < 0.001
St. Petersburg	5,384 342	50 (27.32%)	133 (72.68%)	183	*р* = 0.971
Total	164 210 407	1,089 (27.44%)	2,880 (72.56%)	3,969	

The 2021 registry encompasses a comprehensive dataset consisting of clinical and laboratory information pertaining to a total of 3,969 patients. Among these, 3,563 individuals were actively living, with 46 cases sadly deceased within the same year, and an additional 360 patients not being under observation during this period. Notably, the cumulative count pwCF within the entirety of the Russian Federation, as delineated by the “Program of 14 High-Cost Nosologies” sanctioned by the Ministry of Health of the Russian Federation, is documented at 4,259 individuals.

The comprehensive summary of the 2021 registry is meticulously presented in Table 2. Within the context of this dataset, the average age of patients during the year 2021 was calculated at 14.0 years, with a standard deviation of 9.8 years. The median age stood at 11.9 years, encompassing a range from 6.7 to 19.0 years. Among the pwCF population, there was a slight male predominance, constituting 51.8% of the cohort, while women accounted for the remaining 48.2%.

**TABLE 2:** The main indicators reflecting the organization of care for pwCF and their health status for 2021.

Indicator	2021 y
Total number	3,969
Patient status	
alive, n	3,563
died, n	46
Not observed this year	360
Age, years	
Me (25th – 75th pctl)	11.9 (6.7–19.0)
Mе (IQR)	11.9 (12.4)
Adults (≥18 years), %	27.4
Age at diagnosis, years	
M ± SD	3.1 ± 6.2
Me (25th – 75th pctl)	0.4 (0.1–2.8)
Mе (IQR)	0.4 (2.7)
Diagnosis by neonatal screening	
total, %	53.5
in the reporting year, %	65.8
Age at death (years)	
M ± SD	23.7 ± 10.3
Mе (IQR)	25.8 (12.8)

Mean (M) ± standard deviation (SD), median (Me), Interquartile range (IQR).

Molecular diagnostics were conducted following the clinical guidelines of 2021 (Ministry of Health of the Russian Federation, 2021; Castellani et al., 2018). The approach encompassed a concise three-stage algorithm: (i) analysis of 33 frequent CFTR variants; (ii) Sanger sequencing of the coding regions, intron–exon junctions, 5′- and 3′-UTRs of the *CFTR* gene; and (iii) MLPA analysis for CNV—in DNA samples of CF patients according to the protocol described previously (Petrova et al., 2020). This succinct algorithmic framework ensured a systematic and comprehensive approach to molecular diagnostics, enabling the identification of genetic anomalies associated with cystic fibrosis in patients’ DNA samples.

The variants were analyzed in several databases, namely,: the “RCMG” database on genetics (http://seqdb.med-gen.ru), the CFTR1 database (http://www.genet.sickkids.on.ca/cftr), CFTR2 Base (https://cftr2.org), CFTR-France Base (https://cftr.iurc.montp.inserm.fr/cgi-bin/affiche_var2.cgi), Exome Aggregation Consortium (http://exac.broadinstitute.org), Genome Aggregation Database (http://gnomad.broadinstitute.org), dbSNP (http://www.ncbi.nlm.nih.gov/snp), Exome Variant Server (http://evs.gs.washington.edu/EVS), 1000 Genomes Project (http://browser.1000geno-mes.org/index.html), OMIM (http://www.omim.org), dbVar (http://www. ncbi.nlm.nih.gov/dbvar), Human Gene Mutation Database (http://www.hgmd.cf.ac.uk/ac/in-dex.php), Clin Var (http://www.ncbi. nlm.nih.gov/clin-var), Human Genome Variation Society (http://www.hgvs.org/dblist/dblist.html), DECIPHER (https://decipher).

Statistical data processing was performed using the SPSS software package. Depending on the type of distribution, the mean (M) ± standard deviation (SD) or median (Me) (interquartile range) served as measures of central tendency and dispersion. Statistical processing was performed using the Mann-Whitney test, Pearson chi-square test, Fisher’s exact test, and Kruskal–Wallis test. Linear correlation analysis was used. Differences were considered statistically significant at *p* < 0.05.

The study and the form of informed voluntary consent were approved by the Ethics Committee of the “RCMG” of the Ministry of Education and Science of the Russian Federation on 10 February 2021 (the chairman of the Ethics Committee is Prof. L. F. Kurilo).

## 3 Results

The fluctuation of the birth prevalence index of patients with cystic fibrosis (pwCF) between the years 2007 and 2022 was assessed using data extracted from the RCFPR. The data, as presented in Table 3, details the count of identified patients in each respective reporting year, along with the birth prevalence values calculated in relation to the number of newborns. Notably, the analysis of Table 3 reveals a statistically significant variance in birth prevalence values across the examined years (χ^2^ = 26.48; *p* ≤ 0.05; Degrees of Freedom = 14).

**TABLE 3:** Number of identified patients with CF and birth prevalence values (2007–2022).

Year	Number of newborns in the Russian federation	Number of identified patients with cystic fibrosis	Birth prevalence	Proportion (number of identified patients with cystic fibrosis/Number of newborns in the Russian Federation) (%)
2007	1,297,676	129	1:10,060	0.010
2008	1,417,722	142	1:9,984	0.010
2009	1,444,623	145	1:9,963	0.010
2010	1,742,728	166	1:10,498	0.010
2011	1,654,229	193	1:8,571	0.012
2012	1,863,679	181	1:10,297	0.010
2013	1,802,347	186	1:9,690	0.010
2015	1,897,854	193	1:9,833	0.010
2016	1,810,492	206	1:8,789	0.011
2017	1,632,723	159	1:10,269	0.010
2018	1,632,723	135	1:12,094	0.008
2019	1,401,074	149	1:9,940	0.011
2020	1,436,514	155	1:9,268	0.011
2021	1,402,834	146	1:9,608	0.010
2022	1,306,162	104	1:12,559	0.008
Average			1:10,059	

The calculated average birth prevalence values were determined to be 1 per 10,059 births. This observation signifies the dynamic nature of birth prevalence rates for pwCF throughout the investigated timeframe, indicating the importance of continued monitoring and exploration in this field.

Given the reduced life expectancy of pwCF, we conducted an assessment of CF prevalence within the child population across different federal districts, providing a more realistic representation of this metric (refer to Table 4). Notably, the average prevalence value estimated across the federal districts was found to be 1 per 10,168 children, a figure that aligns closely with the birth prevalence rate of 1 per 10,059. These findings underscore the limited impact of childhood mortality on the overall prevalence of CF.

**TABLE 4:** Prevalence of CF in federal districts among child population.

District	Population size of children	Number of children with CF	Prevalence	Proportion (%)
Central Federal District	7 207 281	805	1:8,953	0.011
Northwest Federal District	2 667 774	263	1:10,144	0.010
Southern Federal District	3 337 319	323	1:10,332	0.010
Volga Federal District	6 021 504	566	1:10,639	0.009
Ural Federal District	2 803 655	245	1:11,443	0.009
Siberian Federal District	3 813 914	362	1:10,536	0.009
Far Eastern Federal District	1 877 643	146	1:12,861	0.008
North Caucasian Federal District	2 654 251	170	1:15,613	0.006
Moscow	2 188 233	285	1:7,678	0.013
St. Petersburg	963 262	133	1:7,243	0.014
Total	33 534 836	3 298	1:10,168	0.010

Our analysis indicates notably low mortality rates for pwCF during childhood. Quantitative evaluation of CF prevalence further underscores these observations, revealing a statistically significant range of prevalence rates across federal districts, spanning from 1 per 7,243 in St. Petersburg to 1 per 15,613 in the North Caucasus Federal District (χ^2^ = 96.10; *p* ≤ 0.05; Degrees of Freedom = 9).

In the subsequent phase, an analysis of molecular genetic data pertaining to the identification of genetic variants in the *CFTR* gene was conducted. The scope of genetic investigation coverage in the year 2021 encompassed a substantial 93.6% of CF patients (see Table 5). Notably, this coverage consisted of 94.4% of children and 91.3% of adults among the cases examined.

**TABLE 5:** Coverage and genetic variant distribution in CFTR gene analysis according to the RCFPR 2021.

Indicator, %	2021 y
Genetic research	
coverage, %	93.6
percentage of identified genetic variants, %	90.5
– two identified genetic variants, %	84.2
– one identified genetic variant, %	12.7
– both genetic variants were not identified, %	3.2
F508del/F508del,%	28.2
F508del/not F508del, %	45.4
Not F508del/not F508del, %	24.9
Complex allele, L467F; F508del	1.5
F508del, allele frequency, %	51.5
СFTRdele2,3, allele frequency, %	6.1
E92K, allele frequency, %	3.5

The cumulative frequency of identified alleles was determined to be 90.5%, with a breakdown of 91.5% for children and 90.2% for adults. Among patients subjected to genetic examination, two distinct variants of the *CFTR* gene nucleotide sequence were discerned in 84.2% of cases, while a single variant was detected in 12.7%. In 3.2% of patients, a genetic variant could not be identified.

Within the subset of patients who underwent genetic analysis, a comparable pattern was observed for the detection of genetic variants. Specifically, two genetic variants of the *CFTR* gene nucleotide sequence were identified in 84.2% of children and 84.3% of adults. Conversely, a single genetic variant was ascertained in 13.6% of children and 10.0% of adults, while a minute subset – 2.2% of children and 5.7% of adults–displayed an absence of detected genetic variants.

Supplementary Table S1 provides an overview of the 233 identified pathogenic variants within the *CFTR* gene, organized in descending order of frequency. Among these variants, 132 have been repeatedly observed. Remarkably, 83 of these variants are not documented within the CFTR2 database; however, they are comprehensively described in publications authored by both Russian and international researchers (Krasovsky et al., 2021).

47 genetic variants are not presented in international CFTR databases—W1282R, 3272-16T>A, A96E, D579Y, G509R, E403D, G1047S, G480S, I175V, D993A, G509V, P205T, T277X, Q493R, R153I, Y569H, −461A->G, −741T->G, c.1584+18672A>G, c.1761del, c.2312delA, c.264_268del, c.353delC, c.3615_3625del, c.3717+1219C>A, c.37dupT, c.3873+4485A>T, c.3983T>A, c.546T>A, c.743+2T>A, C590Y, D572N, F1286S, G1249E, G314R, G509D; E217G,I506T, L1093P, L159S, L233F, L568F, N505H, E1433G, K1468N, F1078I, T604I, V392G. Of the 47 identified rare genetic variants: 18 are severe, 16 are mild variants, 13 are of unclear clinical significance: G480S, I175V, G509V, Q493R, R153I, −461A->G, −741T->G, c.3983T>A, c.546T>A, C590Y, G1249E, L233F, F1078I. In addition to the newly identified variants, clinical significance or pathogenicity has not been established for some of the previously described variants. Previously undescribed variants that are missing in the literature and databases are highlighted in Supplementary Table S2 in grey.

The first 44 genetic variants are major for the Russian Federation and occur with a frequency more often than 0.10%. According to the European Register of pwCF in 2020, compiled by analyzing 49,111 genotypes of pwCF from 39 countries, the allelic frequencies of 18 frequent CF-causing variants in the *CFTR* gene that differ from the Russian Federation were determined: F508del – 60.41% (in the RF – 51.55%), G542%X – 2.75% (in the RF– 1.49%), N1303K – 2.18% (in the RF – 1.52%), G551D – 1.26% (in the RF – 0.04%), W1282%X – 1.07% (in the RF – 1.72%), 2,789+5G->A – 1.07% (in the RF - 0.39%), 3,849+10kbC->T – 1.00% (in the RF – 2.22%), CFTRdele2,3%–0.96% (in the RF – 6.11%), R117H – 0.95% (in the RF – 0.03%), 1717-1G->A – 0.88% (in the RF - 0.05%), R553%X– 0.85% (in the RF – 0.18%), 2183AA->G – 0.71% (in the RF – 0.11%), D1152H – 0.63% (in the RF – 0.12%), 621+1G->T – 0.62% (in the RF – 0.19%), R347P – 0.60% (in the RF – 0.12%), G85E – 0.53% (in the RF – 0.11%), 3272-26A->G – 0.52% (in the RF – 0.05%), R1162%X – 0.51% (in the RF – 0.16%) (ECFSPR Annual report 2020., 2022). The roster of prevalent genetic variants within the Russian Federation is distinctive due to its unique spectrum of pathogenic variants and their frequencies, setting it apart from Europe and other global regions. As follows from the information presented in Table 6, it is evident that there is an observable variation in the frequencies of these significant genetic variants when considering the geographical distribution in different federal districts. The genetic variants F508del and CFTRdele2.3 are less common (*p* < 0.05) in the North Caucasian Federal District, with a higher frequency (*p* < 0.05) of two genetic variants W1282X (13.5) and 1677delTA (28.4). The E92K variant with a higher frequency (*p* < 0.05) was detected in the North Caucasian Federal District (7.3) and Volga Federal District (9.2). This variation highlights the influence of regional factors on the prevalence of these genetic variants.

**TABLE 6:** Frequency of major genetic variants (frequency > 1.0%) in CFTR gene nucleotide sequence across federal districts of RF.

	Central federal district	Northwest federal district	Southern federal district	Volga federal district	Ural federal district	Siberian federal district	Far eastern federal district	North caucasian federal district	Moscow	St. Petersburg	*р*	Frequency in european register
	(A)	(B)	(C)	(D)	(E)	(F)	(G)	(H)	(I)	(J)		
F508del	52.1	56.1	55.3	50.4	53.8	55.4	53.6	21.4	49.3	51.4	*p* < 0.05	60.41
CFTRdele2.3	7.8	4.9	6.4	5.2	4.8	6.4	6.9	1.6	6.9	5.2	*p* < 0.05	0.96
2184insA	2.2	3.0	2.1	—	2.5	1.8	2.0	2.4	1.9	3.6	*p* < 0.05	no data
2143delT	2.6	2.7	2.0	1.7	1.7	1.3	1.8	—	2.8	3.0	*p* > 0.05	no data
G542X	1.8	2.4	1.6	—	1.0	1.8	1.3	—	1.6	2.8	*p* > 0.05	2.75
3,849+10kbC->T	2.4	2.1	3.4	2.7	1.7	1.4	—	1.1	3.5	1.7	*p* > 0.05	1.00
N1303K	1.9	1.8	1.6	1.7	—	1.5	1.5	1.1	1.8	1.9	*p* > 0.05	2.18
W1282X	1.4	1.6	1.6	—	—	1.1	1.5	13.5	2.2	1.4	p < 0.05	1.07
394delTT	—	1.3	—	1.0	1.0	1.2	—	—	—	—	*p* > 0.05	no data
L138ins	1.9	1.2	1.1	2.3	2.9	1.1	—	—	2.3	1.1	*p* > 0.05	no data
E92K	1.8	1.0	1.0	9.2	2.5	2.7	—	7.3	2.7	—	*p* < 0.05	no data
R334W	—	1.0	—	—	—	—	1.0	—	—	1.4	*p* > 0.05	no data
1677delTA	1.3	—	—	—	1.6	—	—	28.4	2.5	—	*p* < 0.05	0.53
L467F; F508del	—	—	—	1.4	1.3	—	—	—	1.0	—	*p* > 0.05	no data
W1282R	—	—	—	—	1.0	—	—	—	—	—	*p* > 0.05	no data
R1066C	—	—	—	—	—	1.2	—	1.4	—	—	*p* > 0.05	no data
S1159F	—	—	—	—	—	—	—	1.6	—	—	*p* > 0.05	no data
S1196X	—	—	—	—	—	—	—	1.1	—	1.4	*p* > 0.05	no data
A96E	—	—	—	—	—	—	—	1.4	—	—	*p* > 0.05	no data
3821delT	—	—	—	—	—	—	—	1.1	—	1.4	*p* > 0.05	no data

The horizontal axis shows the *p* values for the estimated prevalence of each genetic variant in the federal districts of the country.

**FIGURE 1:**
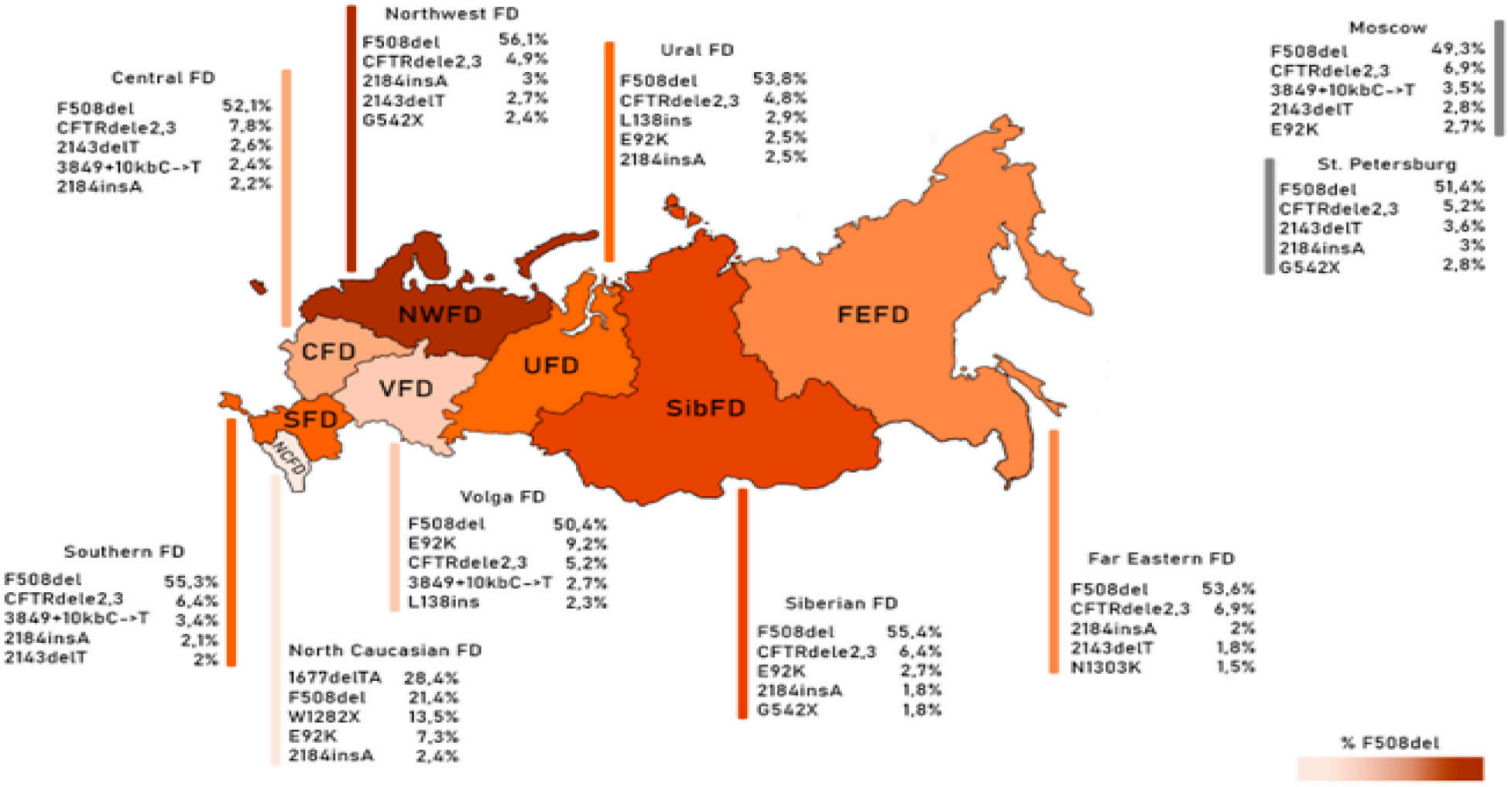
Frequency of the first five variants of the CFTR gene nucleotide sequence in different federal districts.

Guided by these disparities identified across European countries, the subsequent phase involved an in-depth analysis of the spectrum and frequencies of major genetic variants, defined as those occurring with a frequency surpassing 1.0%, across the Federal Districts of the Russian Federation. The outcomes of this analysis are succinctly summarized in Table 6 and Figure 1.

As deduced from the information presented in Table 6, it is evident that there exists an observable variation in the frequencies of these significant genetic variants when considering the geographical distribution across the various Federal Districts. This variation underscores the impact of regional factors on the prevalence of these genetic variants.

**FIGURE 2:**
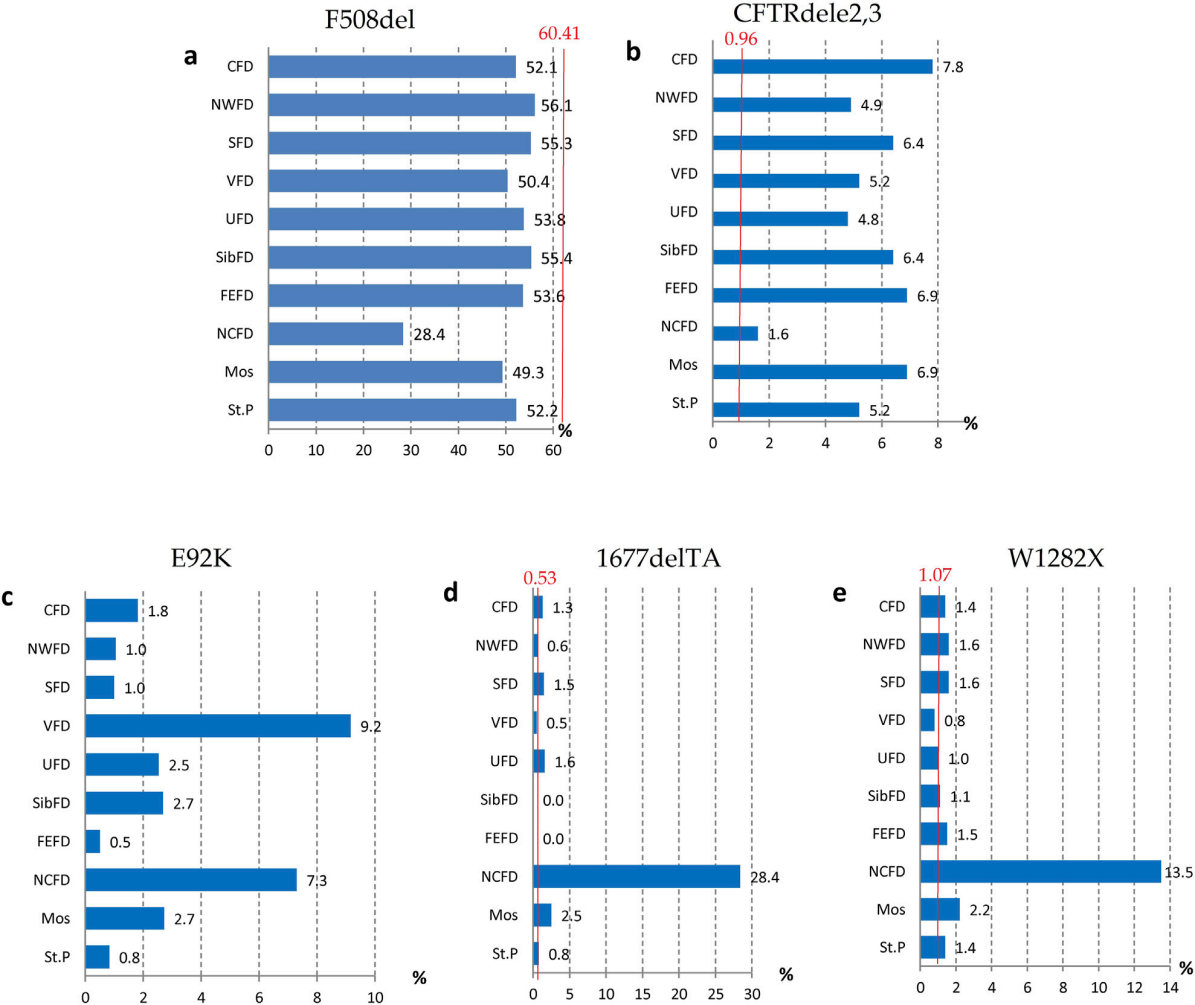
Variation of frequencies of genetic variants in different federal districts of the Russian Federation. **(A)** Frequency of the genetic variant of the F508del *CFTR* gene; **(B)** Frequency of the genetic variant of the CFTRdele2,3 *CFTR* gene; **(C)** Frequency of the genetic variant of the E92K *CFTR* gene; **(D)** Frequency of the genetic variant of the 1677delTA *CFTR* gene; **(E)** Frequency of the genetic variant of the W1282X *CFTR* gene. Note: CFD - Central Federal District; NWFD - Northwest Federal District; SFD - Southern Federal District; VFD - Volga Federal District; UFD - Ural Federal District; SibFD - Siberian Federal District; FEFD - Far Eastern Federal District; NCFD - North Caucasian Federal District; Mos–Moscow; St.P - St. Petersburg The red line indicates the frequency in the European registry.

The genetic landscape of Europe highlights the prevalence of F508del as the most frequent genetic variant within the *CFTR* gene. This variation exhibits distinct frequencies across different countries, with its prevalence ranging from under 5% in Georgia and Armenia to over 82% in Albania (Festini et al., 2008). Figure 2A illustrates the frequency distribution of the pathogenic F508del variant within the Federal Districts of the Russian Federation. Notably, the average frequency in Russia rests at 51.55%, showcasing a range that spans from 56.1% in the Northwestern Federal District—dominantly inhabited by the Slavic population—to 28.4% in the North Caucasian Federal District.

Another prevalent variation in the Russian Federation is the CFTRdele2,3 deletion, commonly referred to as the “Slavic” deletion. The average frequency of this variation is 6.11%, with fluctuation from 1.6% in the North Caucasian Federal District to 7.8% in the Central Federal District, as depicted in Figure 2B. In European countries, the average frequency of this genetic variant stands at 0.96%, with its peak prevalence observed in Belarus at 10.9%.

The most significant variation among the prevalent genetic variants was identified in pathogenic variants E92K, 1677delTA, and W1282X, particularly pronounced in the North Caucasian and Volga Federal Districts—polyethnic regions renowned for their diverse populations (Petrova et al., 2021). Figure 2C illustrates the frequency distribution of the E92K nucleotide sequence variant within the Federal Districts. Nationally, the average allele frequency for E92K was determined as 3.46%. The E92K mutation emerged as the third most frequent pathogenic variant in the Russian Federation. Notably, it exhibited a higher prevalence of 9.2% in the Volga region and 7.3% in the North Caucasus Federal District, while being comparatively rarer in other Federal Districts (Kondratyeva et al., 2023; Petrova et al., 2019).

Concerning the genetic variant 1677delTA, it exhibited high allelic frequency solely in the North Caucasian Federal District at 28.4%. However, variants with frequencies exceeding 1.0% were found in the Central, Southern, and Ural Federal Districts as illustrated in Figure 2D. Notably, the variant 1677delTA was most prevalent among Chechen individuals at 67.3%, and it was also detected in other small ethnic groups in the North Caucasus region (Petrova et al., 2019). This variant, originally identified in Georgian individuals with a frequency of around 25%, is common in Mediterranean countries, albeit at significantly lower frequencies (e.g., Bulgaria (2.1%), Romania (0.8%), Greece (0.7%), Turkey (4.1%), Cyprus (approximately 6%)) (World Health Organization, 2004).

**FIGURE 3:**
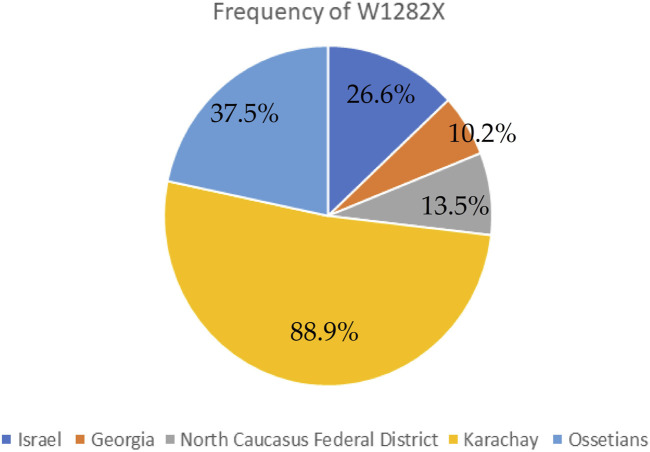
Frequency of W1282X in different countries and certain populations.

The genetic variant W1282X (Figure 2E and Figure 3), originating in theMiddle East, dominates in Israel with an allelic frequency of 22.6%, especially prominent among Ashkenazi Jews. It’s also notably present in Georgia at 10.2% (Kerem et al., 1995). Within the North Caucasus Federal District, the W1282X variant exhibited an average frequency of 13.5%, with particular prominence in certain populations like the Karachay (88.9%) and Ossetians (37.5%) (Petrova et al., 2016; Petrova et al., 2020).

The identification of these genetic variants holds pivotal implications for the potential of pathogenetic therapy for pwCF. Among the 233 genetic variants identified, the majority (116) belong to class I disorders, with 6 in class II, 5 in class III, 11 in class IV, 11 in class V, and 1 in class VI. The class remains undefined in 84 variants.

Determining the mutation class and genotype type (severe or mild) based on age is also essential for enabling pathogenetic therapy. The first eleven variants within the nucleotide sequence of the *CFTR* gene in both children and adults are provided in Table 7.

**TABLE 7:** The allelic frequencies of the most common CFTR genetic variants among children and adults (in descending order).

N	Children (<18 years)			Adults (≥18 years)			
	Genetic variant *CFTR* gene	Class	Frequency, %	Genetic variant *CFTR* gene	Class	Frequency, %	p
1	F508del	II	52.94	F508del	II	47.74	*p* < 0.001
2	CFTRdele2,3	VII	6.29	CFTRdele2,3	VII	5.63	*p* = 0.298
3	E92K	IV-V	3.14	E92K	IV-V	4.33	*p* = 0.014
4	1677delTA	I	2.81	1677delTA	I	0.70	*p* < 0.001
5	W1282X	I	2.00	W1282X	I	0.96	*р* = 0.002
6	2143delT	I	1.95	2143delT	I	2.06	*p* = 0.755
7	2184insA	I	1.86	2184insA	I	2.16	*p* = 0.397
8	G542X	I	1.54	G542X	I	1.36	*p* = 0.559
9	L138ins	IV	1.51	L138ins	IV	2.01	*p* = 0.130
10	N1303K	II	1.49	N1303K	II	1.61	*p* = 0.707
11	3,849+10kbC->T	V	1.19	3,849+10kbC->T	V	5.03	*p* < 0.001
12	L467F; F508del	II	1.01	L467F; F508del	II	0.0	*p* < 0.001

The frequency of homozygotes, heterozygotes according to F508del and genotypes without F508del among children and adults is shown in Table 8.

**TABLE 8:** The frequencies of homozygotes and heterozygotes for the F508del variant, as well as genotypes without F508del among children and adults.

Group	F508del/F508del	F508del/not F508del	Not F508del/not F508del
Children, %	29.9	46.5	23.6
Adult, %	23.7	48.0	28.3
p	*p* < 0.001	*p* = 0.412	*p* = 0.04

**FIGURE 4:**
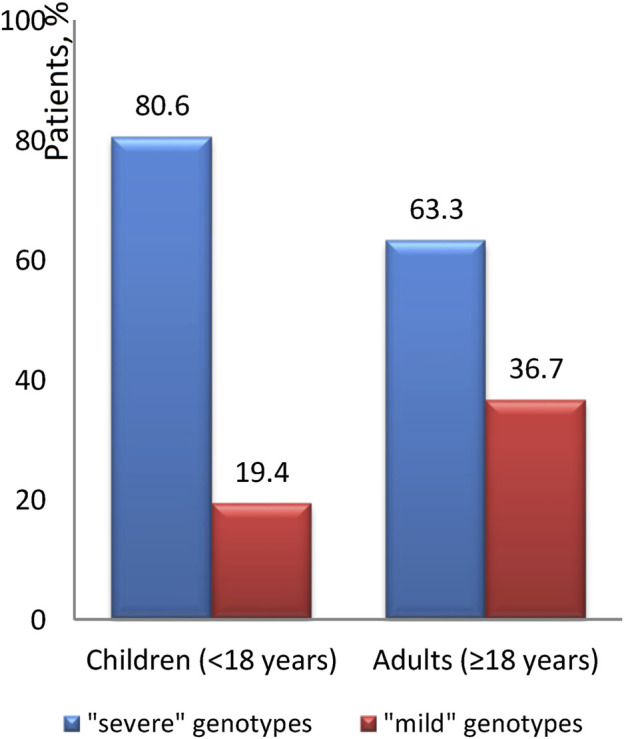
The ratio of the “severity” of genotypes depending on age.

**FIGURE 5:**
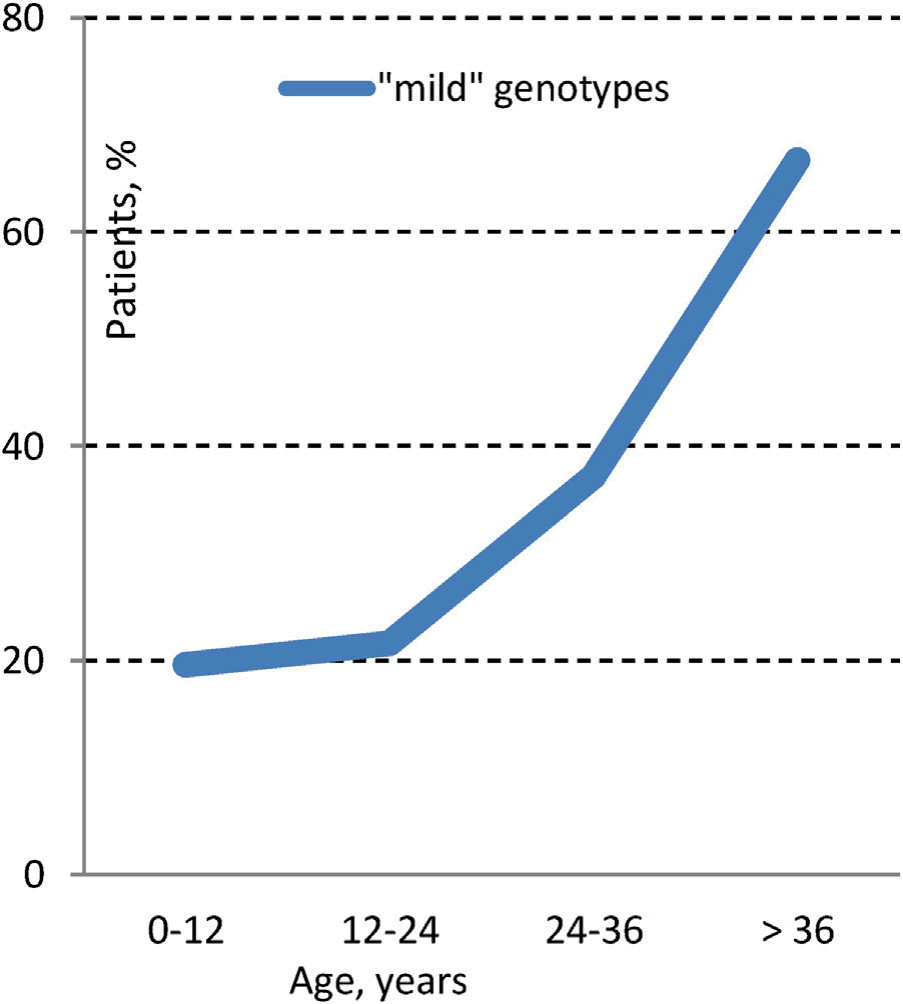
Distribution of “mild” genotypes in different age groups.

The occurrence of the “mild” genotype was identified in 24.1% of patients. The distribution of these “mild” genotypes in relation to age is graphically illustrated in Figures 4, 5. We found that ‘severe’ genotypes are predominant among both children and adults - amounting to 80.6% before the age of 18, and slightly diminishing to 63.3% after the age of 18.

Upon closer examination of age-related distinctions, it's revealed that the “mild” genotype was detected in 19.6% of patients under 12 years of age and a notable 66.7% of patients over 36 years of age. Interestingly, the average age of patients with the “mild” genotype is 17.7 ± 12.4, with a median of 14.2 (18.5). Conversely, the average age of patients with the “severe” genotype is 12.9 ± 8.6, with a median of 11.2 (10.7).

Of the 3,714 patients who underwent DNA diagnosis, one or both variants were found only in 3,598 patients (97%) (Table 9). Of the 3,598 patients, F508del/F508del was found in 30.5%, F508del/not F508del in 47.0%, and not F508del/not F508del in 22.5% (Table 9). In 7.5% of patients, 1 variant in the genotype is among 177 FDA-approved variants for ETI, 6.9% of patients can receive ETI because at least one variant is expected to respond to ETI, in 9.8% both variants are not suitable for treatment with targeted drugs due to the absence of CFTR protein (Table 9).

**TABLE 9:** Distribution of genetic variants with possible efficacy of targeted therapy according to the data of the Registry 2021.

Genotype	N	%
F508del/F508del	1,097	30.5
F508del/not F508del	1,693	47.0
Not F508del/not F508del	808	22.5
At least one of 177 FDA-approved variant	234	7.5
Approved for HEMT treatment (at least one non FDA-approved variant predicted to respond to ETI)	215	6.9
Not approved for HEMT treatment	306	9.8

In the Russian Federation, the drug Lumacaftor/Ivacaftor is registered in 2020, and Elexacaftor/Tezacaftor/Ivacaftor + Ivacaftor in 2023. The frequency of modulators use was 6.47% in 2021 (Table 10).

**TABLE 10:** Frequency of use of different types of CFTR modulators in children and adults in 2021.

CFTR-modulators, %	All	Children	Adults
Ivacaftor	0.40	0.10	1.19
Lumacaftor/Ivacaftor	2.72	3.06	1.84
Tezacaftor/Ivacaftor + Ivacaftor	0.28	0.07	0.83
Elexacaftor/Tezacaftor/Ivacaftor + Ivacaftor	3.07	2.53	4.50
Total	6.47	5.76	8.36

In 2023 (as of 01.09.2023), 1,504 children (52.2% of children with CF) are provided with targeted therapy, of which 1,046 (69.55%) receive ETI therapy, taking into account the severity of the children’s condition. Lumacaftor/Ivacaftor is received by 458 children (30.45%). The number of adults on targeted therapy is 350 patients (32,2%).

The efficacy of targeting drugs not specified in the instructions for use in RF was proved by Forskolin-induced swelling (FIS) test on intestinal organoids in patients with variants: E92K, N1303K, L138ins, G1047S, 3272-16T>A in the genotype (Table 11).

**TABLE 11:** Pathogenic variants in the patient’s genotype that are not included in the instructions for the drugs, but there is data from a study of effectiveness on Forskolin-induced swelling (FIS) test using intestinal organoids.

Pathogenic variant	
Е92К (Kondratyeva et al., 2023) c.274G>A p.Glu92Lys	lumacaftor + ivacaftor
Е92К (Kondratyeva et al., 2023) c.274G>A p.Glu92Lys	ivacaftor + tezacaftor + elexacaftor and ivacaftor (later included in the instructions)
L138ins (Melyanovskaya et al., 2020) c.411_412insCTA p.Leu138dup	lumacaftor + ivacaftor
L138ins (Melyanovskaya et al., 2020) c.411_412insCTA p.Leu138dup	ivacaftor + tezacaftor + elexacaftor and ivacaftor
3272-16T>A (Kondratyeva et al., 2021) c.3140–16T>A No protein name	ivacaftor + tezacaftor + elexacaftor and ivacaftor
N1303K (DeStefano et al., 2018; Ensinck et al., 2022) c.3909C>G p. (Asn1303Lys)	lumacaftor + ivacaftor
N1303K (Amelina et al., 2021; Gona-Hoepler et al., 2023; Cohen-Cymberknoh et al., 2023; Sadras et al., 2023; Burgel et al., 2023) c.3909C>G p. (Asn1303Lys)	ivacaftor + tezacaftor + elexacaftor and ivacaftor
G1047S c.3139G>A p. (Gly1047Ser)	ivacaftor + tezacaftor + elexacaftor and ivacaftor

In patients with variants S466X; R1070Q, L467F; F508del, c.1083G>A, c.831G>A, c.1513A>C, c.1329_1350del in the genotype, ineffective treatment with targeted drugs was proved by FIS (Table 12).

**TABLE 12:** Pathogenic variants in the patient’s genotype that did not respond to targeting drugs in the Forskolin-induced swelling (FIS) test using intestinal organoids.

Pathogenic variant	CFTR modulator
S466X; R1070Q (Krasnova et al., 2023)	lumacaftor + ivacaftor
S466X; R1070Q (Krasnova et al., 2023)	ivacaftor + tezacaftor + elexacaftor and ivacaftor
L467F; F508del (Kondratyeva et al., 2022a; Kondratyeva et al., 2022b)	lumacaftor + ivacaftor
L467F; F508del (Kondratyeva et al., 2022a; Kondratyeva et al., 2022b)	ivacaftor + tezacaftor + elexacaftor and ivacaftor
c.1083G>A (Kondratyeva et al., 2019)	ivacaftor + tezacaftor + elexacaftor and ivacaftor
c.831G>A (Kondratyeva et al., 2020)	ivacaftor + tezacaftor + elexacaftor and ivacaftor
c.1513A>C (Melyanovskaya et al., 2021)	ivacaftor + tezacaftor + elexacaftor and ivacaftor
c.1329_1350del (Kondratyeva et al., 2024)	ivacaftor + tezacaftor + elexacaftor and ivacaftor

## 4 Discussion

Since 2011, a meticulous RCFPR has been methodically cultivated within the Russian Federation, adhering to the stringent standards set forth by the European register. This comprehensive database encompasses a range of clinical indicators, therapeutic interventions, and, notably, an in-depth section dedicated to genetic epidemiology and molecular genetic diagnostics. This segment provides valuable insights into disease prevalence, the gamut of genetic variations within Russian patients, the availability of DNA diagnostics (including *CFTR* gene sequencing), and the potential for pathogenetic therapies.

The primary objective of this publication is threefold: to present the birth prevalence of pwCF, exhibiting variations over the years; to outline the spectrum and frequency of *CFTR* gene disorders within the Russian patient population; and to underscore the distinct aspects characterizing the Russian cohort in comparison to the European registry, as well as the role of the registry in planning the availability of CFTR modulators and identifying a group of patients with rare pathogenic variants who may receive ETI if at least one variant is expected to respond to ETI after forskolin testing on intestinal organoids (Forskolin-induced swelling (FIS) assay using intestinal organoids).

These differences encompass noteworthy disparities, such as the proportion of patients aged over 18, which constitutes a mere 27.4% in contrast to the 53% observed in the European Cystic Fibrosis Society Patient Registry (ECFSPR) for the year 2020 (Orenti et al., 2022). An imminent increase in this statistic is anticipated post-2025, attributed to the transition of children identified through neonatal screening to the adult category, and the implementation of CFTR modulators.

Although the Russian cohort boasts a commendable genetic diagnostic coverage of 93%, as compared to Europe’s aggregate of 99%, the share of identified alleles is noted at 90.5%. It's noteworthy that 12.7% of Russian patients possess an incomplete genetic diagnosis—a figure higher than the 5.48% documented among European patients in 2020. Furthermore, a subset of 3.2% of Russian patients remain in the dark about both alleles. This discrepancy primarily stems from the disparate accessibility to comprehensive DNA diagnostics across different regions of the vast country, with limited availability of extended genetic examination. Another distinctive hallmark of the Russian national pwCF register lies in the extensive diversity of genetic variants identified. A total of 233 variants have been documented, exhibiting frequencies ranging from 0.01% to 51.5%, with 47 of these variants remaining uncharted within international genetic databases. Notably, the clinical significance of certain variants remains undetermined, contributing to the intricate tapestry of genetic diversity within the Russian population. Substantial disparities exist in the allelic frequency of the most prevalent variants within the *CFTR* gene. For instance, the widely recognized F508del variant, which is identified on average in 60.4% of European cases and can reach as high as 80% in certain countries, demonstrates a frequency of 51% of alleles within the Russian Federation. Additionally, the proportion of homozygotes for F508del in Russia (28%) significantly trails behind the European average of 40%. This variation highlights distinct genetic characteristics within the Russian population.

Another noteworthy pathogenic variant, G542X, which holds second place in the European register at 2.75%, occupies the eleventh position in the Russian register at 1.49%. Conversely, the E92K variant, prevalent in Russia at 3.46%, is notably absent from the European list of 18 variants with an allelic frequency of up to 0.5%. This divergence underscores the unique genetic landscape within Russia, contributing to variations in the prevalence of specific pathogenic variants.

The spectrum of frequent genetic variants further diverges based on Federal Districts, as it was presented in Table 6 and Figure 1. The Russian patient population showcases an extensive array of genetic variants within the *CFTR* gene, with a notable 116 variants belonging to class I variants—a distinctive hallmark of the genetic diversity in the region.

Starting from 2021, CFTR modulator therapy has become accessible in the Russian Federation for patients, significantly elevating the importance of genetic diagnosis. In 2023, more than 1,850 patients received CFTR modulator therapy. Rare genetic variants have been studied using the ICM method and FIS assay to improve patient coverage of targeted therapy as in other countries (Dreano et al., 2023).

As insights accumulate regarding the potential impact of complex alleles—comprising two or more variants in the cis position—there’s an emerging recognition of their influence not only on the progression of cystic fibrosis but also on the efficacy of targeted therapies tailored to this condition (Baatallah et al., 2018).

This genetic variant exploration among Russian patients specifically highlights the prevalence of the complex allele [L467F; F508del], a combination that has demonstrated a tangible impact on the effectiveness of targeted ivacaftor/lumacaftor therapy in F508del homozygotes. Russian research into the prevalence of this specific cis combination of pathogenic variants and a number of rare variants (E92K, N1303K, L138ins, G1047S) has prompted a reevaluation of clinical recommendations concerning the administration of this particular CFTR modulator (Table 12) (Petrova et al., 2022; Kondratyeva et al., 2022b; Sondo et al., 2022).

In the context of the modern capabilities for disease-modifying therapy, it's imperative to extend efforts towards comprehending the clinical significance and pathogenicity of newly identified variants. Furthermore, exploring the feasibility of implementing targeted therapies for individuals carrying these variants is a pivotal undertaking.

## 5 Conclusion

In conclusion, the Russian population of pwCF is characterized by a rich diversity of variants within the *CFTR* gene. This diversity is marked by a preponderance of severe variants, some of which are rare and distinct from those observed in other populations. In light of contemporary advancements in disease-modifying therapies, it is imperative to focus on the comprehensive evaluation of the clinical significance and pathogenicity of these new genetic variants. Equally vital is exploring the feasibility of employing targeted therapies for individuals who carry these specific variants.

As the medical landscape continues to evolve, understanding the genetic intricacies that contribute to disease progression and the efficacy of therapeutic interventions is of paramount importance. The dynamic interaction between genetic variations and modern treatment modalities underscores the necessity of ongoing research efforts aimed at enhancing the quality of care for individuals with cystic fibrosis.

This comprehensive analysis sheds light on the intricate genetic mosaic that defines the cystic fibrosis landscape in Russia, emphasizing the critical role of genetic research in guiding therapeutic strategies. For a more comprehensive grasp of the nuances within the Russian population’s genetic makeup, their clinical implications, and the ongoing advancements in cystic fibrosis research, referring to the original source or document is highly recommended.

## Data Availability

The data analyzed in this study is subject to the following licenses/restrictions: All patient data should be anonymized. Requests to access these datasets should be directed to Elena Kondratyeva, elenafpk@mail.ru.
